# Src and Abl hijack the Robo1 pathway

**DOI:** 10.18632/oncotarget.122

**Published:** 2010-07-15

**Authors:** Christian Naus

**Affiliations:** Life Sciences Institute, The University of British Columbia, Vancouver, BC, Canada

Src and Abl are non-receptor tyrosine kinases that promote tumor cell migration. This hallmark of malignant cell growth is required for tumor cell invasion and metastasis. However, mechanisms by which Src and Abl augment these events are not well defined. In this report [1], Khusial et al. have elegantly shown that Src activates Abl, which stabilizes the Robo1 receptor to enhance tumor cell migration. Robo1 signaling has been implicated in tumor progression and is an enticing target for chemotherapeutic drugs. Robo1, upon binding to its ligand Slit2, recruits a number of Kinases, GTPase activating proteins, and Rho GTPases to rearrange the actin cytoskeleton and promote cell migration. The authors have demonstrated that Src activates Abl, and that Abl associates with Robo1 to stabilize its activity and expression in transformed cells. Importantly, the authors have also shown that siRNA and Kinase blockers can reduce Abl expression and activity to dampen Robo1 activity and transformed cell migration. Moreover, they demonstrate that siRNA and a monoclonal antibody against Robo1 (R5 Ab) can suppress the migration of transformed cells in the face of robust Src and Abl kinase activity. These studies suggest that a variety of reagents may be used to target a pathway at different steps to suppress tumor cell migration.
